# Quantile regression in genomic selection for oligogenic traits in autogamous plants: A simulation study

**DOI:** 10.1371/journal.pone.0243666

**Published:** 2021-01-05

**Authors:** Gabriela França Oliveira, Ana Carolina Campana Nascimento, Moysés Nascimento, Isabela de Castro Sant'Anna, Juan Vicente Romero, Camila Ferreira Azevedo, Leonardo Lopes Bhering, Eveline Teixeira Caixeta Moura

**Affiliations:** 1 Department of Statistics, Federal University of Viçosa, Viçosa, Minas Gerais, Brazil; 2 Center of Ruber Tree and Agroforestry Systems, Agronomy Institute (IAC), Votuporanga, São Paulo, Brazil; 3 AGROSAVIA, The Colombian Agricultural Research Corporation, Mosquera, Colômbia; 4 Department of General Biology, Federal University of Viçosa, Viçosa, Minas Gerais, Brazil; 5 Empresa Brasileira de Pesquisa Agropecuária—Embrapa Café, Brasília, DF, Brazil; Graduate University of Advanced Technology, Kerman Iran, ISLAMIC REPUBLIC OF IRAN

## Abstract

This study assessed the efficiency of Genomic selection (GS) or genome‐wide selection (GWS), based on Regularized Quantile Regression (RQR), in the selection of genotypes to breed autogamous plant populations with oligogenic traits. To this end, simulated data of an F_2_ population were used, with traits with different heritability levels (0.10, 0.20 and 0.40), controlled by four genes. The generations were advanced (up to F_6_) at two selection intensities (10% and 20%). The genomic genetic value was computed by RQR for different quantiles (0.10, 0.50 and 0.90), and by the traditional GWS methods, specifically RR-BLUP and BLASSO. A second objective was to find the statistical methodology that allows the fastest fixation of favorable alleles. In general, the results of the RQR model were better than or equal to those of traditional GWS methodologies, achieving the fixation of favorable alleles in most of the evaluated scenarios. At a heritability level of 0.40 and a selection intensity of 10%, RQR (0.50) was the only methodology that fixed the alleles quickly, i.e., in the fourth generation. Thus, it was concluded that the application of RQR in plant breeding, to simulated autogamous plant populations with oligogenic traits, could reduce time and consequently costs, due to the reduction of selfing generations to fix alleles in the evaluated scenarios.

## Introduction

In mid-2019, the world population reached 7.7 billion inhabitants and a further rise to 9.7 billion by 2050 is estimated [1]. Thus, more food must be produced to feed this population, although agricultural areas are increasingly limited and concerns about the negative environmental impacts of food production are growing [2, 3].

Since the Green Revolution in the 1960s, which caused a boost in the production potential of several crops, it is generally expected that plant breeding efforts will be able to secure the required yield gains [4]. The productivity of coffee trees, for example, has increased considerably, and one of the main reasons is the use of improved cultivars. In Brazil, coffee cultivars that were released and are still in use, e.g., “Mundo Novo”, are 240% more productive than introduced varieties [5]. Plant breeding programs, aside from focusing on higher yields, require the improvement of several other traits [6], e.g., the development of plants with a more appropriate architecture for higher plant density and mechanical management, better resistance and tolerance to biotic and abiotic stresses, adaptation to and stability in different cultivation environments, and a higher fruit and grain quality [7–11].

To meet the growing producer, consumer and market demand, a complex, continuous and dynamic breeding process is required [4], resulting in costly long-term projects for the development of superior cultivars. The developmental period of an improved cultivar of a perennial species can be over 25 years [12] and for annual species approximately 12 years. Thus, the search for procedures capable of providing superior genotypes in less time and, consequently, at a lower cost, has been intensified [4, 13, 14].

With a view to reducing the time demand and increasing selection accuracy, Meuwissen et al. [15] proposed the genome-wide selection (GWS). This kind of selection uses direct DNA information based on molecular markers to predict the genomic estimated breeding value (GEBV) of an individual, which is a measure used to select the best individuals, according to their merit within the population. The main advantage of GWS, compared to phenotypic selection, is that the GEBV of individuals whose phenotypes were not yet collected can be estimated, thus resulting in a reduced generation interval and an increase in genetic gain [16–18].

The possibilities of applying GWS in autogamous plant breeding have been described in the literature. According to Heffner et al. [19], the prediction accuracy of GWS was superior to phenotypic selection in wheat. In simulated scenarios to improve oligogenic traits in *Coffea Arabica*, with different population densities and sizes, Romero [20] tried to determine the generation in which a favorable allele is fixed. As a result, the author observed that in small populations (as commonly used in breeding programs, e.g., for coffee), favorable alleles were fixed in the sixth generation (*F*_6_), while in large populations, fixation occurred in the fifth generation (*F*_5_). The GWS was also successfully applied in other crops, such as rice [21], oats [22] and barley [23].

An alternative and still little explored methodology for GWS studies is Quantile Regression (QR) [24]. Such methodology, unlike traditional methods based on averages, allows to adjust regression models throughout the distribution of the dependent variable, does not require assumptions about the distribution of the error and is robust to outliers. Parameter estimation is based on the weighted absolute errors method [25]. To deal with dimensionality problems in GWS studies, which are common in the marker matrix, Li and Zhu [26] proposed the Regularized Quantile Regression (RQR). The use of RQR in a GWS study was proposed by Nascimento et al. [27], in order to estimate GEBV for different quantiles of the phenotype of interest [28, 29]. In their study, Nascimento et al. [27] used RQR to estimate GEBV from simulated data with scenarios with different skewness levels in the phenotype distribution. The results of the RQR were compared to those of the BLASSO (Bayesian Least Absolute Shrinkage and Selection Operator) method, and the authors observed a lower mean square error of the former. The results indicated the viability of this alternative for GWS analysis, even in scenarios without skewness of the phenotype distribution. The approaches RQR and BLASSO were also used by Santos et al. [30] to estimate the genetic merit in pigs for asymmetric traits related to the pig carcass, and observed equally or more accurate results by RQR than BLASSO for all evaluated traits.

In spite of the interesting and promising results, RQR has not yet been evaluated throughout an entire breeding process, considering the reproductive system of a plant species. Thus, this study evaluated whether the use of RQR in GWS, for simulated data of autogamous plants with oligogenic traits, at different selection and heritability levels, allows the fixation of favorable alleles in earlier generations than the commonly used GWS methodologies. The results of the predictive capacity, mean and genotypic variance obtained by RQR were compared with traditional methods of genomic selection, specifically with RR-BLUP and BLASSO.

## Material and methods

### Population simulation

For this study, a 1040 cM genome was simulated, using software GENES [31], with markers spaced 1 cM apart, with eight linkage groups, resulting in a total of 1048 markers [32]. Oligogenic traits controlled by four loci were simulated, located in four different linkage groups, with uniform effect and absence of dominance and epistasis.

The *F*_1_ population was established by crossing contrasting parents, thus generating gametes for the formation of the *F*_2_ population, consisting of 625 individuals. Once the base genome was generated, genotypic values and three sets of phenotypic values were simulated, at heritability levels (*h*^2^) of 0.10, 0.20 and 0.40. To determine the genotypic values (*vg*_*i*_), the following equation was used:

$$vg_{i}=\mu +a_{i}+d_{i},$$ (1)

where *vg*_*i*_ is the genotypic value of individual i; *μ* the genotype population mean (here *μ* = 1.0); *a*_*i*_ the additive effect of individual i, with $a_{i}=\sum_{k=1}^{4}\rho_{k}\alpha_{k}$, where *ρ*_*k*_ = 2.5 is the effect of the favorable allele with the same contribution to the whole locus *k*; *α*_*k*_ the contribution of locus *k* (1, 0 or -1 for genotypic classes AA, Aa and aa, respectively); and *d*_*i*_ is the dominance effect, assumed to be null in this study (*d*_*i*_ = 0).

The phenotypic values (*vf*_*i*_) were determined by the following equation:

$$vf_{i}=vg_{i}+\varepsilon_{i},$$ (2)

where *vf*_*i*_ is the phenotypic value of individual *i*; *vg*_*i*_ the genotypic value of individual *i*; *ε*_*i*_ the environmental effect generated according to a normal distribution, where mean and variance are compatible with the specific trait heritability ($\varepsilon_{i}∼N(0,\sigma_{e}^{2})$), with $\sigma_{e}^{2}=\frac{\sigma_{g}^{2}(1-h^{2})}{h^{2}}$, where $\sigma_{g}^{2}$ is the genetic variance [33]. The phenotypic and genotypic simulated data sets are freely accessible at https://zenodo.org/record/4292736#.X8BDrmhKjIU.

The advanced generations *F*_3_, *F*_4_, *F*_5_ *and F*_6_ were obtained from *F*_2_ as base generation by selfing. The individuals with the highest GEBV obtained by an adjusted GWS model in *F*_2_ were selected. The number of selected individuals depends on the selection intensity. The selection/simulation process of the progenies was repeated until the sixth generation. The generations *F*_3_ to *F*_6_, with 200 individuals [34], were generated from the genotype of the selected individuals, simulating a selfing process. The *F*_3_ to *F*_6_ populations were simulated using software R [35].

### Genomic prediction

Based on the simulated *F*_2_ population, it was stipulated that 80% of the individuals would belong to the estimation population and 20% to the validation population. The genomic genetic values of the individuals were estimated by RQR [26] based on different quantiles (0.10, 0.50 and 0.90), using RR-BLUP [15] and BLASSO [36]. Two selection intensities (10% and 20%) and three heritability levels (*h*^2^ = 0.10, 0.20 *and* 0.40) were considered, and each evaluated scenario was simulated 30 times. For all evaluated methods, the general GWS model was considered [15]:

$$y_{i}=\mu +\sum_{j=1}^{1040}x_{ij}g_{j}+e_{i},$$ (3)

where *y*_*i*_ is the i^th^ observation of phenotype *y* (*i* = 1,2,…,625); μ the overall mean; *g*_*j*_ the effect of the j^th^ marker (*j* = 1,2,…,1040); *x*_*ij*_ are the elements of the incidence matrix of marker j in individual i, with parameterization 1, 0 and −1; and *e*_*i*_ is the i^th^ observation of the random error $e_{i}∼N(0,\sigma_{e}^{2})$.

The parameters of model (2) were estimated by three methodologies: RQR, at three quantiles (τ = 0.10, 0.50 and 0.90), BLASSO and RR-BLUP.

In RQR, the marker effects are computed by solving the following optimization problem [26]:

$$\hat{g}=argmin\left\{\sum_{i=1}^{n}\rho_{\tau }(y_{i}-\mu -\sum_{j=1}^{p}x_{ij}g_{j})+\lambda \sum_{j=1}^{p}\left|g_{j}\right|\right\},$$ (4)

where $\sum_{j=1}^{p}\left|g_{j}\right|$ is the sum of the absolute values of the regression coefficients, λ the penalty parameter, *n* = 625, *p* = 1040 and *ρ*_*τ*_(.), called “check function” by Koenker and Bassett (1978), and defined by:

$$\rho_{\tau }\left(y_{i}-\mu -\sum_{j=1}^{p}x_{ij}g_{j}\right)=\left\{ \begin{array}{c} \tau \left(y_{i}-\mu -\sum_{j=1}^{p}x_{ij}g_{j}\right),se\;y_{i}-\mu -\sum_{j=1}^{p}x_{ij}g_{j}\geq 0\\ (\tau -1)\left(y_{i}-\mu -\sum_{j=1}^{p}x_{ij}g_{j}\right),otherwise \end{array}\right.$$

in this study, *τ* = 0.1; *τ* = 0.5 and *τ* = 0.9.

Note that, in the RQ, the coefficients are estimated from the minimization of the weighted sum of the vertical distances between the observed and estimated values [25]. For that, linear programming algorithms are used [37, 38]. One of the methods used to estimate these coefficients is the Simplex Method. For details on the Simplex Method, please consult Koenker [38].

After estimating the regression coefficients (marker effects), for the three quantiles considered (*τ* = 0.1; *τ* = 0.5 and *τ* = 0.9), the GEBV of the i^th^ individual, based on quantile *τ* (*GEBV*_*i*_(*τ*)), was calculated by the following equation:

$$GEBV_{i}(\tau )={\hat{y}}_{i}(\tau )=\sum_{j}^{p}x_{ij}{\hat{g}}_{j}(\tau ),$$ (5)

where $\hat{g}(\tau )$ is the estimated effect of the j^th^ SNP marker based on quantile *τ* (*τ* = 0.1; *τ* = 0.5 e *τ* = 0.9). The GEBVs were also determined by BLASSO and RR-BLUP, according to the equation:

$$GEBV_{i}={\hat{y}}_{i}=\sum_{j}^{p}x_{ij}{\hat{g}}_{j},$$ (6)

where ${\hat{g}}_{j}$ is the effect of the j^th^ SNP marker, estimated by the two said methods.

According to the recommendation of Santos et al. [30], the penalty parameter *λ* of RQR was defined as half the penalty parameter resulting from the BLASSO method.

To compare the analyzed methodologies, the predictive capacity $\left(r_{y\hat{,y}}\right)$ of the methods was calculated, which is the correlation coefficient between the observed phenotypic (*y*) and the estimated genomic value ($\hat{y}$) in each generation. The genotypic means and variances in each generation were also determined.

Based on Eq 1, with *μ* = 1.0 and $a_{i}=\sum_{k=1}^{4}\rho_{k}\alpha_{k}=10$, it can be said that favorable alleles in a given generation are fixed when the genotypic mean of a population reaches 11 and variance zero.

### Computational aspects

The calculations and estimates were performed in the R program [35]. The function used to estimate the regression parameters at the three quantiles was rq of the quantreg package [39]. The regression coefficients were estimated by RR-BLUP using the mixed.solve function of package rrBLUP [40]. The Bayesian models were adjusted with the bglr function in package BGLR [41], with 100,000 iterations for the MCMC (Markov chain Monte Carlo) algorithms, of which 10,000 were discarded as burn-in, to ensure chain heating and a thin of 5. Convergence analysis was performed based on the criteria proposed by Raftery and Lewis [42] and Heidenberg and Welch (1983) [43].

## Results and discussion

**Fig 1:**
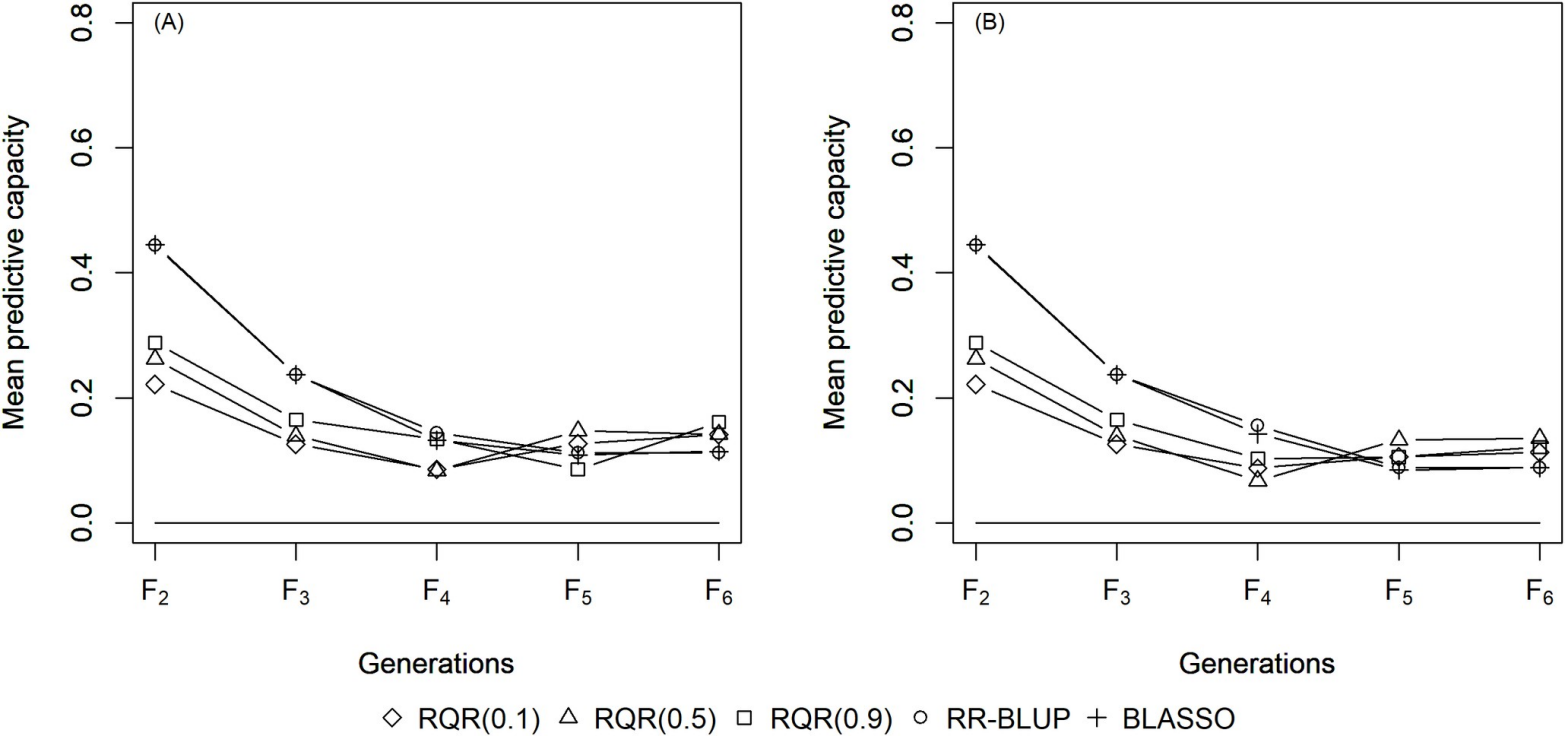
Average predictive capacity (y-axis) of the models evaluated over five generations (x-axis). Considering a heritability of 0.10 and two selection intensities. (A) SP = 10%; (B) SP = 20%.

**Fig 2:**
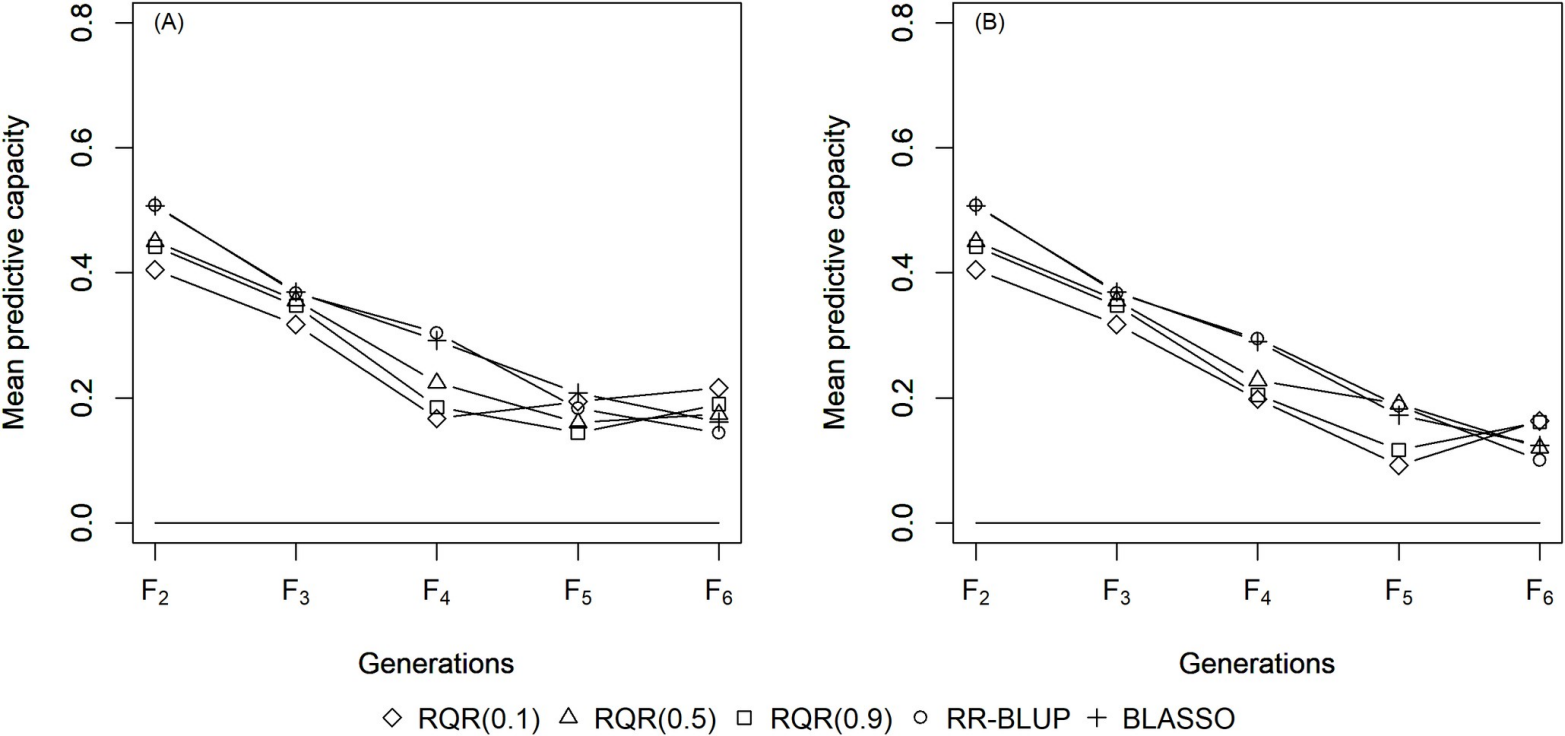
Average predictive capacity (y-axis) of the models evaluated over five generations (x-axis). Considering a heritability of 0.20 and two selection intensities. (A) SP = 10%; (B) SP = 20%.

**Fig 3:**
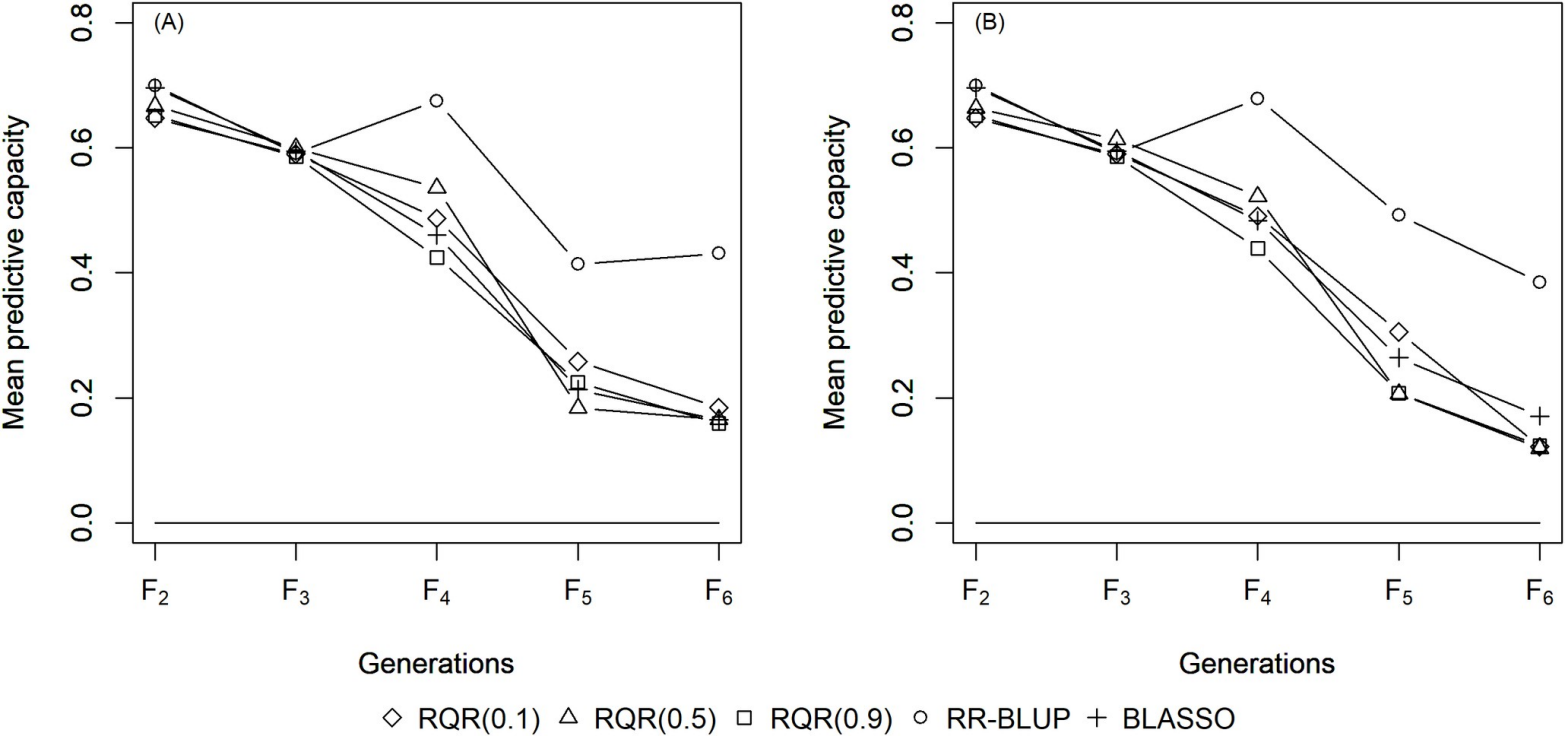
Average predictive capacity (Y axis) of the models evaluated over five generations (x axis). Considering a heritability of 0.4 and two selection intensities. (A) SP = 10%; (B) SP = 20%.

The mean estimates of the predictive capacity (PC) in scenarios with heritability of 0.10 varied between 0.20 (RQR (0.10)) and 0.45 (BLASSO and RR-BLUP) in generation *F*_2_ (Fig 1); between 0.40 (RQR (0.10)) and 0.5 (BLASSO and RR-BLUP) at heritability 0.20 (Fig 2); and between 0.65 (RQR (0.10) and RQR (0.90)) and 0.70 (RR- BLUP) in the scenarios with heritability of 0.40 (Fig 3). In general, PC rises as heritability increases (Figs 1–3). This result was already expected, since traits with higher heritability are less affected by environmental variation, facilitating the breeding process [44].

Over the generations, the PC estimates decreased, to values close to zero in *F*_6_ in several scenarios (Figs 1–3). This result can be explained by the fact that the model to predict GEBV in the F_3_ –F_6_ generations was adjusted in F_2_. Specifically, since selection occurs over generations, the allele frequency of the initial generation changes, which leads to a reduction in the marker-QTL linkage disequilibrium (LD). Over the generations, Sant’Anna et al. [33] observed a drop in LD, which is reflected in the predictive capacity of the model for autogamous populations. In allogamous species on the other hand, LD is dissipated by advancing a single generation, resulting in a low efficiency of GS procedures based on models adjusted in previous populations.

There was an increase in means over the generations and a decrease in the genotypic variances to values close to zero from the third generation onwards, for all evaluated methods (Table 1 and S1–S3 Figs). These results are in line with the theory of quantitative population genetics, which states that in response to directional selection, the allele frequency of traits with few major-effect loci changes rapidly, inducing a phenotypic response [45]. In this way, the population mean increases since the allele value is positive in the simulation process.

**Table 1:** Means and mean genotypic variances (n = 30) of five generations in response to selection by five predictive methods, based on three heritability levels (0.10, 0.20 and 0.40) and two selection intensities (10 and 20%).

h^2^	Methods	SP	F3		F4		F5		F6	
			GM	GV	GM	GV	GM	GV	GM	GV
0.10	BLASSO	10%	6.44±0.30	9.03±1.35	7.78±0.98	7.66±2.22	9.14±1.33	5.56±4.26	10.45±0.96	1.46±1.69
		20%	5.76±0.20	10.55±0.90	7.73±0.48	8.48±1.52	9.09±0.98	5.74±2.83	10.15±1.06	2.24±1.99
	RRBLUP	10%	6.48±0.31	9.05±1.30	7.89±0.79	8.14±2.10	9.45±1.03	4.82±2.41	10.24±1.22	1.78±2.38
		20%	5.82±0.21	10.34±1.02	7.81±0.59	8.46±1.47	9.14±0.98	5.90±2.92	10.31±0.92	2.30±2.93
	RQR (0.10)	10%	4.19±1.05	11.97±2.44	5.91±1.57	9.92±2.97	7.89±1.93	6.10±3.80	8.54±2.19	2.21±3.01
		20%	3.69±0.93	14.00±2.01	5.44±1.13	11.24±2.08	7.73±1.68	7.04±2.92	8.39±1.72	4.50±3.57
	RQR (0.50)	10%	4.34±0.82	12.07±2.59	6.47±1.49	9.14±3.26	8.20±1.86	6.16±4.64	9.42±2.06	2.01±2.95
		20%	3.80±0.74	13.63±2.30	5.88±1.20	10.93±2.47	8.39±1.57	7.16±5.19	9.74±1.63	2.24±2.78
	RQR (0.90)	10%	4.78 ±0.83	11.12±2.42	6.71 ±1.20	8.83±3.57	7.96±2.11	5.15± 4.53	8.76±2.67	2.40±2.90
		20%	4.47±0.80	12.02±2.01	6.39±1.20	9.64±3.10	7.85±1.57	6.90±3.53	9.09±2.23	3.25±3.39
0.20	BLASSO	10%	7.49±0.29	6.02±0.79	9.95±0.40	3.04±0.92	10.75±0.37	0.82±1.18	10.95±0.16	0.21±0.58
		20%	6.90±0.20	7.38±0.66	9.70±0.35	3.65±0.79	10.68±0.27	1.13±0.89	10.90±0.23	0.37±0.79
	RRBLUP	10%	7.47±0.30	6.21±0.91	9.97±0.41	2.94±0.90	10.73±0.27	0.94±0.84	10.80±0.43	0.64±1.35
		20%	6.90±0.18	7.37±0.68	9.70±0.19	3.86±0.65	10.65±0.28	1.30±1.18	10.88±0.26	0.40±0.79
	RQR (0.10)	10%	6.40±0.75	7.42±1.90	7.85±1.14	5.84±1.78	9.01±1.55	3.86±2.28	9.25±2.00	1.85±2.53
		20%	5.84±0.59	10.02±1.69	7.86±0.81	6.63±1.49	9.02±1.22	4.72±1.94	10.02±1.50	1.78±1.98
	RQR (0.50)	10%	6.69±0.50	6.95±1.65	9.41±0.78	4.38±1.74	10.72±0.55	1.07±1.94	10.98±0.14	0.11±0.59
		20%	6.36±0.64	8.24±2.13	9.12±0.94	5.35±2.53	10.56±0.72	1.53±2.18	10.92±0.26	0.34±1.02
	RQR (0.90)	10%	6.32±0.62	7.68±1.78	8.01±1.25	5.99±2.08	9.26±1.61	3.29±2.61	10.30±1.42	0.88±1.80
		20%	6.09±0.49	8.67±1.84	8.08±1.01	6.92±2.14	9.25±1.37	4.29±2.80	10.20±1.26	4.29±2.69
0.40	BLASSO	10%	8.01±0.16	4.79±0.60	10.47±0.23	1.71±0.67	10.84±0.25	0.54±0.81	10.99±0.03	0.03±0.13
		20%	7.31±0.15	6.76±0.62	10.18±0.21	2.70±0.63	10.82±0.20	0.63±0.68	10.95±0.10	0.20±0.37
	RRBLUP	10%	7.98±0.20	4.95±0.69	10.45±0.26	1.83±0.87	10.87±0.21	0.45±0.66	10.94±0.15	0.20±0.50
		20%	7.32±0.13	6.64±0.49	10.18±0.26	2.60±0.73	10.43±0.17	0.62±0.62	10.94±0.21	0.20±0.62
	RQR (0.10)	10%	7.97±0.24	4.81±0.73	10.56±0.30	1.47±0.97	10.95±0.16	0.21±0.67	10.96±0.23	0.16±0.86
		20%	7.37±0.23	6.28±0.64	10.27±0.29	2.35±0.83	10.95±0.17	0.20±0.49	10.97±0.12	0.12±0.51
	RQR (0.50)	10%	8.29±0.22	3.95±0.65	10.86±0.21	0.50±0.69	10.95±0.18	0.16±0.57	11.00±0.00	0.00±0.00
		20%	7.54±0.17	5.93±0.64	10.68±0.23	1.11±0.76	10.96±0.09	0.14±0.31	10.98±0.10	0.09±0.40
	RQR (0.90)	10%	7.74±0.30	5.45±1.12	10.17±0.39	2.68±1.28	10.64±0.39	1.28±1.34	10.77±0.62	0.64±1.31
		20%	7.15±0.25	6.91±0.92	9.98±0.44	3.32±1.59	10.54±0.41	1.73±1.56	10.73±0.64	0.80±1.65

h^2^: heritability; SP: selection intensity; GM: genotypic mean; GV: genotypic variance; RQR: regularized quantile regression; BLASSO: Bayesian Lasso. Genotypic means and variances in F_2_ were 1.10 ± 0.00 and 12.19 ± 0.00, respectively, in all tested scenarios.

The results of fixation or non-fixation of favorable alleles were different in the evaluated scenarios. In scenarios with trait heritability of 0.10, the RQR (τ = 0.1) with a selection intensity of 10% failed to fix the favorable alleles until the sixth generation, as it did not reach the genotypic mean 11 (8.54 ± 2.19) (Table 1). When selection was based on the models RQR (τ = 0.1) or BLASSO, with a selection intensity of 20%, the favorable alleles were not fixed until the sixth generation, as the genotypic variance did not reach zero by these methods (4.50 ± 3.57 and 2.24 ± 1.99, respectively) (Table 1). For the other methods, even in low-heritability scenarios, the favorable alleles could be fixed until the sixth generation (Table 1). According to Goddard [46], the speed at which a population increases or decreases the level of an allele depends on its initial frequency. Thus, the greatest difficulty in fixing alleles in a low-heritability scenario may be due to the greater environmental effect that affects the estimation of GEBV, making it even more difficult to select individuals with the desired alleles than in the other scenarios, where the disturbing environmental effect is lower.

For the scenarios with a trait heritability of 0.20 and regardless of the selection intensity, the tested methods allowed the fixation of favorable alleles until the sixth generation, except for RQR (τ = 0.90), at a selection intensity of 20% (Table 1). Selection based on the BLASSO models, at an intensity of 10%, and on RQR (τ = 0.50), at intensities of 10 and 20%, reached fixation in F_5_ (Table 1).

Moreover, at a heritability level of 0.40, RQR (τ = 0.50) at a selection intensity of 10% allowed the establishment of favorable alleles as early as in the fourth generation, with a genotypic mean of 10.86 ± 0.21 and genotypic variance of 0.50 ± 0.69, while the other methods allowed allele fixation in the fifth or sixth generation (Table 1). With these results, there was a reduction of one (h^2^ = 0.20) or two generations (h^2^ = 0.40) in the fixation process of favorable alleles. The reduction of generations in a plant breeding program is decisive in view of the savings in terms of time, efforts and costs. In coffee for example, one selection generation lasts on average six years [47], i.e., by this technique, the breeding process can be considerably reduced, thus reducing the time required to develop genetically superior genotypes and, consequently, save costs.

Although the BLASSO and RR-BLUP methods had the highest predictive capacity in *F*_2_ in all evaluated scenarios, the results in relation to favorable allele fixation were equal to or lower than by RQR (0.50).

Generally speaking, the breeding process by RQR can be equal to or faster than by the standard GS methodologies. Although to date little explored in breeding, the RQR method has been shown to be very promising for genomic selection and association studies, in both plant and animal breeding [27–30, 48]. In this study, RQR (τ = 0.50) fixed the favorable alleles in the fourth generation (*F*_4_) in the scenario with a heritability of 0.4 and selection intensity of 10%. The efficiency of RQR, in contrast with the traditional methods, based on conditional means, can be explained by the possibility of fitting models at different levels (quantiles) of the phenotype distribution, and consequently making a more thorough study of the phenomenon of interest possible [24]. Specifically, for highly skewed phenotypic distributions, the results of quantile models that allow a quantile fitting far from the mean are interesting. In an evaluation of quantiles 0.25 and 0.75 for right- and left-skewed distributions, respectively, Nascimento et al. [27] and Barroso et al. [28] observed that these models have a higher predictive capacity and lower mean square errors than the traditional GS methodologies, respectively.

In this study, since the data were generated assuming a normal (symmetrical) distribution, better results were expected from mean- or median-based methodologies. However, the best results were based on medians, which may be related to the rarity of occurrence, both in simulated and in practical processes, of a perfectly symmetrical distribution. Thus, a median-based methodology such as RQR (τ = 0.50) can better describe the functional relationship between the dependent and explanatory variables and is robust to outliers, in cases of symmetry deviations in the phenotype distribution [25, 38].

## Conclusions

The use of Regularized Quantile Regression models proved effective in genomic selection studies, for allowing an accelerated development of superior genotypes in relation to traditional GS methodologies. Among the simulated conditions, the configuration of Regularized Quantile Regression (τ = 0.50), at a heritability of 0.40 and selection intensity of 10% was the most efficient, since favorable alleles could be fixed more quickly, as early as in the fourth generation.

## Supporting information

S1 Fig Means (blue lines) and mean genotypic variances (red lines) of the models evaluated over five generations. Considering heritability 0.10 and two selection intensities (SP). (A) SP = 10%; (B) SP = 20%.
(TIF)

S2 Fig Means (blue lines) and mean genotypic variances (red lines) of the models evaluated over five generations. Considering heritability 0.20 and two selection intensities (SP). (A) SP = 10%; (B) SP = 20%.
(TIF)

S3 Fig Means (blue lines) and mean genotypic variances (red lines) of the models evaluated over five generations. Considering heritability 0.40 and two selection intensities (SP). (A) SP = 10%; (B) SP = 20%.
(TIF)
