# Supplementary figures and images for: Quantile regression in genomic selection for oligogenic traits in autogamous plants: A simulation study
Source: PLoS One. 2021 Jan 5;16(1):e0243666. doi: 10.1371/journal.pone.0243666 (PMC7785117; doi:10.1371/journal.pone.0243666)

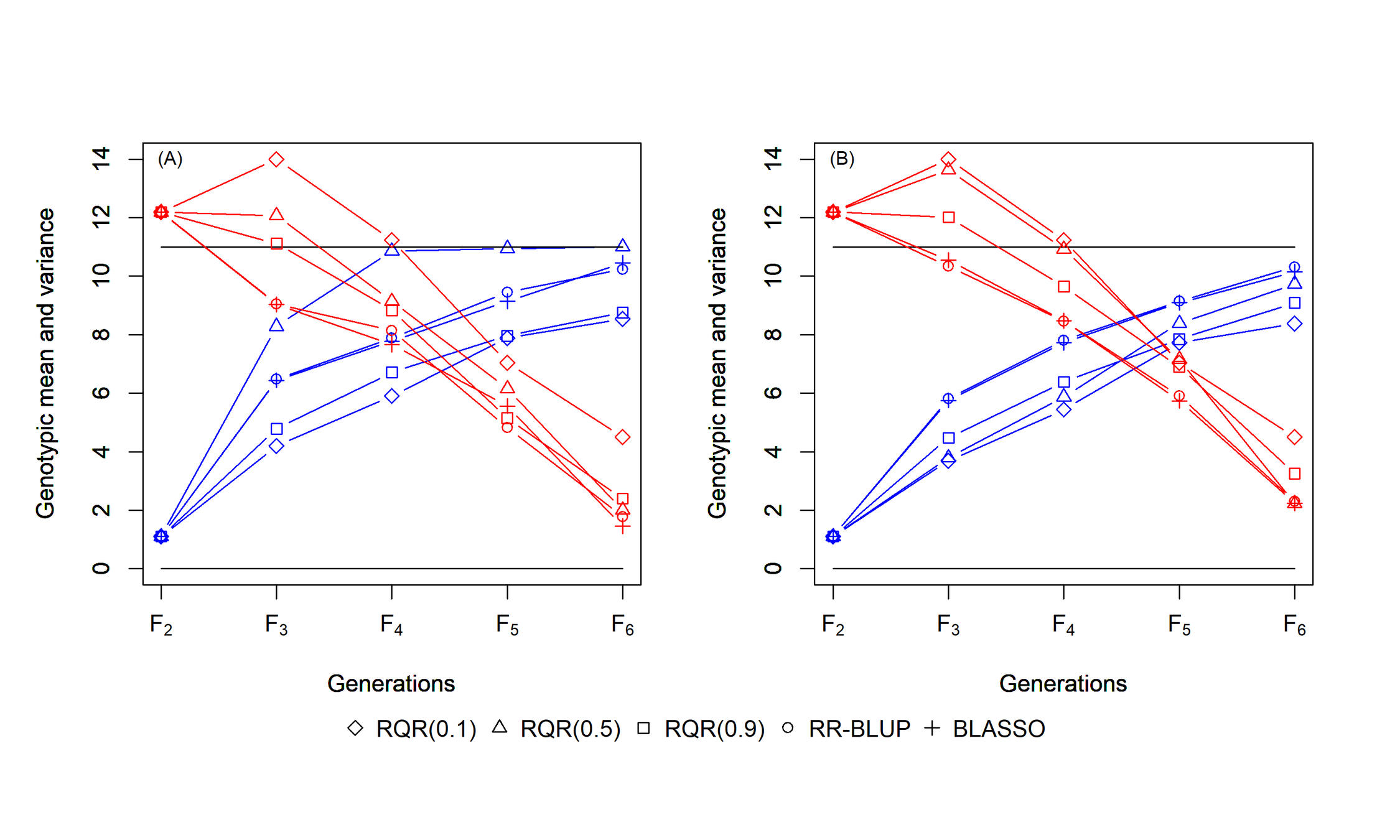

Supplement: S1 Fig — Considering heritability 0.10 and two selection intensities (SP). (A) SP = 10%; (B) SP = 20%. (TIF) [file pone.0243666.s001.tif]

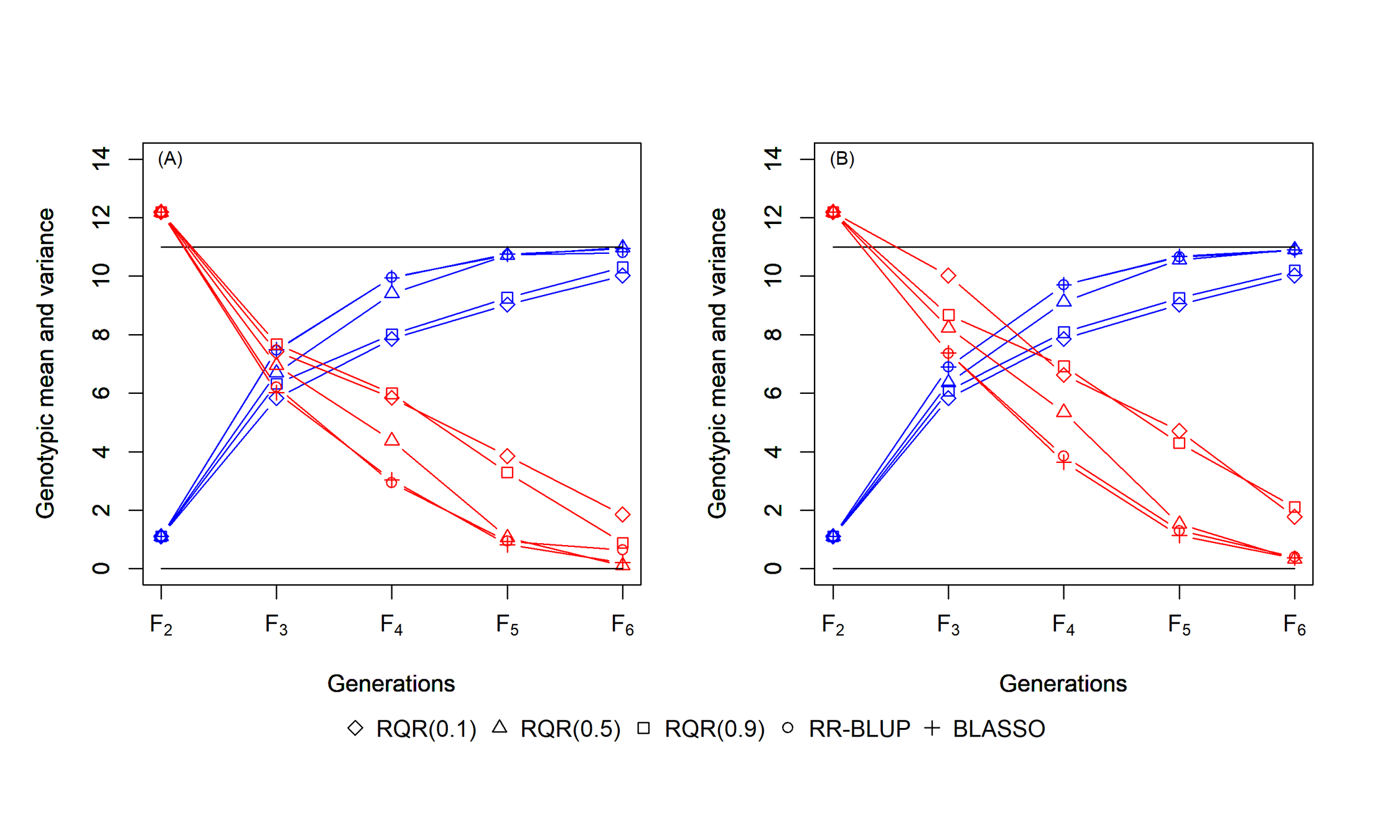

Supplement: S2 Fig — Considering heritability 0.20 and two selection intensities (SP). (A) SP = 10%; (B) SP = 20%. (TIF) [file pone.0243666.s002.tif]

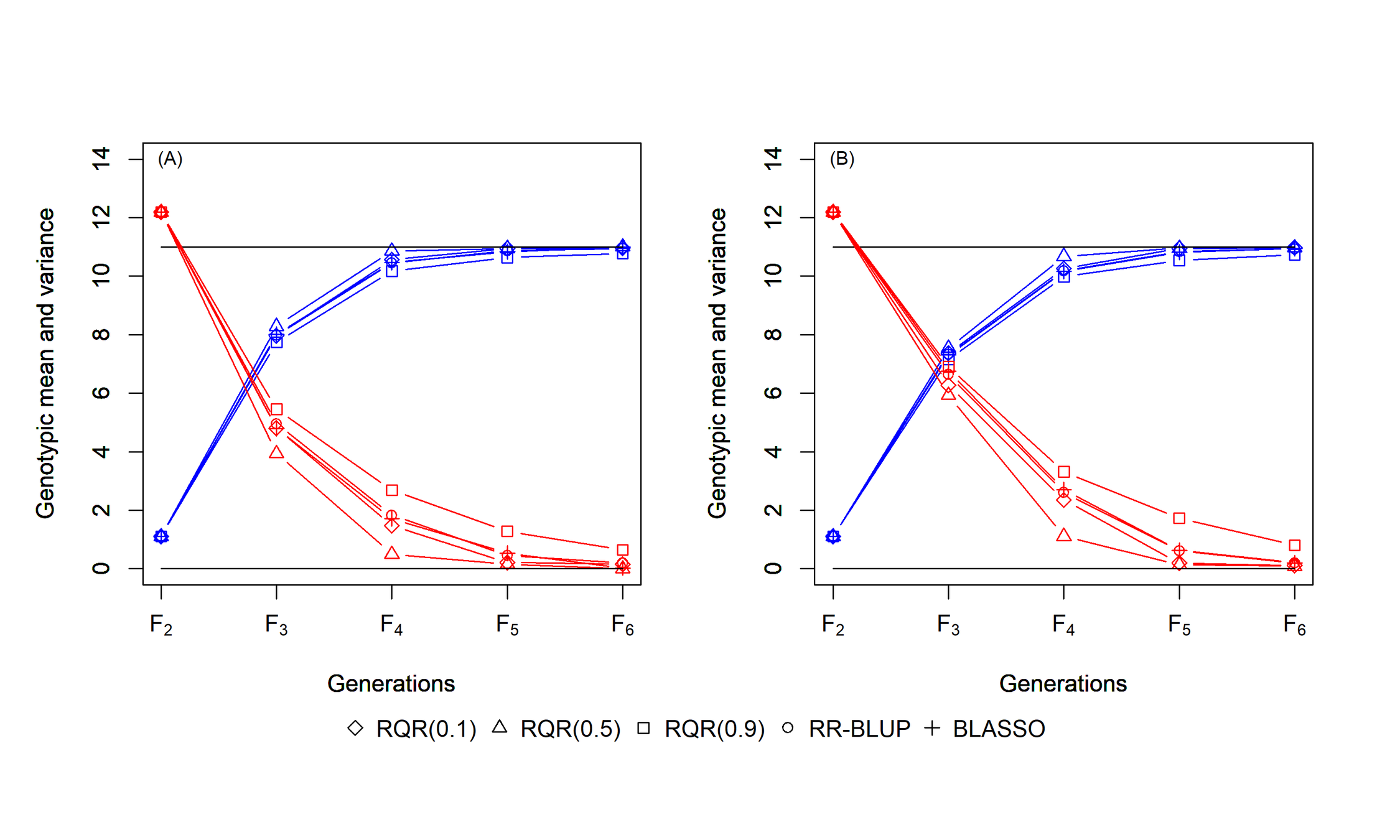

Supplement: S3 Fig — Considering heritability 0.40 and two selection intensities (SP). (A) SP = 10%; (B) SP = 20%. (TIF) [file pone.0243666.s003.tif]
